# Quantifying the duration of the preclinical detectable phase in cancer screening: a systematic review

**DOI:** 10.4178/epih.e2022008

**Published:** 2022-01-03

**Authors:** Sandra M. E. Geurts, Anne M. W. M. Aarts, André L. M. Verbeek, Tony H. H. Chen, Mireille J. M. Broeders, Stephen W. Duffy

**Affiliations:** 1 Department of Medical Oncology, GROW-School for Oncology and Developmental Biology, Maastricht University Medical Center, Maastricht, The Netherlands; 2 Radboud Institute for Health Sciences, Department for Health Evidence, Radboud University Medical Center, Nijmegen, The Netherlands; 3 Institute of Epidemiology and Preventive Medicine, National Taiwan University, Taipei, Taiwan; 4 Dutch Expert Centre for Screening, Nijmegen, The Netherlands; 5 Wolfson Institute of Preventive Medicine, Barts and The London School of Medicine and Dentistry, Queen Mary University of London, London, UK

**Keywords:** Systematic review, Preclinical detectable phase, Sojourn time, Early detection of cancer, Breast neoplasms, Colorectal neoplasms

## Abstract

**OBJECTIVES:**

The aim of this study was to provide an overview of published mathematical estimation approaches to quantify the duration of the preclinical detectable phase (PCDP) using data from cancer screening programs.

**METHODS:**

A systematic search of PubMed and Embase was conducted for original studies presenting mathematical approaches using screening data. The studies were categorized by mathematical approach, data source, and assumptions made. Furthermore, estimates of the duration of the PCDP of breast and colorectal cancer were reported per study population.

**RESULTS:**

From 689 publications, 34 estimation methods were included. Five distinct types of mathematical estimation approaches were identified: prevalence-to-incidence ratio (n=8), maximum likelihood estimation (n=16), expectation-maximization algorithm (n=1), regression of observed on expected (n=6) and Bayesian Markov-chain Monte Carlo estimation (n=5). Fourteen studies used data from both screened and unscreened populations, whereas 19 studies included only information from a screened population. Estimates of the duration of the PCDP varied between 2 years and 7 years for breast cancer in the Health Insurance Plan study (annual mammography and clinical breast examinations in women aged 40-64 years) and 2 years and 5 years for colorectal cancer in the Calvados study (a guaiac fecal occult blood test in men and women aged 45-74 years).

**CONCLUSIONS:**

Different types of mathematical approaches lead to different estimates of the PCDP duration. We advise researchers to use the method that matches the data available, and to use multiple methods for estimation when possible, since no method is perfect.

## INTRODUCTION

Cancer screening programs aim to detect cancer precursors or cancers at an early stage, in order to prevent cancer death by timely treatment. Treatment for cancer precursors or cancers in their preclinical phase is generally more effective than treatment upon clinical symptoms [1]. The duration of the preclinical detectable phase (PCDP), also referred to as the sojourn time, can be considered the window of opportunity for screening to detect a cancer early. This duration is correlated with the sensitivity of the screening test and the underlying cancer incidence. A screening test that can detect very early stages of cancer is associated with a longer PCDP duration than a test that detects a higher number of cancers but at a more advanced stage. As such, both the PCDP duration and the sensitivity of the screening test are central to the determination of the screening interval [1].

Quantifying the duration of the PCDP is challenging because it is not directly observable. We know when an individual cancer is diagnosed, but we do not know for how long prior to diagnosis it was in the PCDP. The PCDP duration can be seen as a function of tumor growth, often expressed as volume doubling time [2,3]. However, volume doubling times are difficult to estimate since once a cancer is diagnosed it is excised or otherwise treated, and its natural growth cannot be observed thereafter. Therefore, a number of mathematical approaches have been developed to estimate the duration of the PCDP based on data from cancer screening programs, including rates of detection of cancer at first and subsequent screens and the incidence rates of interval cancers [4-11].

Since an overview of these approaches and their underpinnings is not available, we have conducted a systematic review that can help researchers choose the mathematical approach that best suits their data. As there is no preferred mathematical estimation approach for the PCDP duration *a priori*, we attempted to investigate the impact of the mathematical approach used on the actual estimate by studying its application to breast and colorectal cancer screening program data. Specificity is another important attribute of a screening test, but since this is readily estimable from empirical false-positive data, without complex modeling, we focused on the duration of the PCDP and sensitivity.

## MATERIALS AND METHODS

### Literature search and study selection

We performed a systematic search to identify studies that described new or adapted mathematical models designed to estimate the duration of the PCDP in cancer screening. The search strategy was composed of 3 parts—(1) cancer; (2) screening; and (3) PCDP, sojourn time, or lead time (Supplementary Material 1)—and was performed in PubMed and Embase on February 8, 2018. Only primary studies in English or Dutch were included. Methods measuring tumor volume doubling time and using microsimulations were excluded. Finally, the references of the included studies were searched for relevant missing articles.

### Data extraction

For each study, 2 researchers (AMWMA and SMEG) extracted details on the mathematical approach, data source, cancer type, and model assumptions with arbitration by a third researcher (SWD) where needed. Next, the studies were grouped by the mathematical estimation approach and classified per data source (screened and/or unscreened population). Per study, the following attributes were collected: screening rounds (first and/or subsequent), data used for estimation (screen-detected and/or interval cancers), the underlying incidence of cancer, assumed distribution(s) of the PCDP duration and/or lead time, confidence intervals or standard error, and test sensitivity. The underlying incidence of diagnosed cancer is sometimes considered to be equal to the rate of transition from the no-disease state to preclinical cancer [12], although in the presence of overdiagnosis, this assumption is problematic. When data are available on people not invited to the screening program, the underlying incidence can be observed from this comparison group. Otherwise the underlying incidence either needs to be estimated within the model, observed from registry data, or not included. Test sensitivity was assumed to be 100%, estimated within the mathematical model or observed from the literature, or not included at all.

Information was sought on whether the assumed model of natural history allowed tumors to regress (i.e., allowed the possibility of a decrease in the size, extent, or even presence of cancer) and whether the model corrected for length bias and/or overdiagnosis. Definitions of the screening terms used are summarized in Table 1.

### Applications in breast and colorectal cancer screening

To investigate the effects of the different approaches on PCDP estimates, we compared estimates of the PCDP duration for breast and colorectal cancer between studies with different mathematical approaches, but based on the same data set (screening test, population and age range). From the included studies, screening program details including data used, age range, and test sensitivity were collected.

### Ethics statement

Not applicable, a systematic review does not require institutional review board approval.

## RESULTS

From 689 publications identified in the search, 33 were included in this review, describing 34 methods (Supplementary Material 2). From these, 5 distinct mathematical approaches to estimate the PCDP duration were identified: prevalence-to-incidence ratio [6,13-19], maximum likelihood estimation [7,12,18,20-32], expectation-maximization algorithm [33], regression of observed on expected [8,11,34-37], and Bayesian Markov-chain Monte Carlo estimation (Table 2) [18,30,38-40]. Fourteen methods required data on a screened and an unscreened population [6,7,13-17,20,21,23,33-35,39], whereas 19 obtained estimates using only information from a screened population [8,11,12,18,19,22,24-32,36-38,40]. The 5 mathematical estimation approaches are described below; details concerning the model assumptions in the individual studies per type of mathematical approach are reported in Table 3 and Supplementary Material 3. The algebraic development of the 5 mathematical approaches, with examples, is described in Supplementary Material 4.

### Prevalence-to-incidence ratio

The prevalence-to-incidence ratio is the simplest way to estimate the PCDP duration and was applied in 8 of the included studies [6,13-19]. This model is based on the well-established relationship, wherein disease prevalence is equal to the product of the incidence of the disease and the average disease duration (in this case, preclinical disease duration) [42]. The attraction of this approach is its simplicity, as the estimation can be carried out with closed form algebra. A disadvantage is that in its simplest form it assumes 100% sensitivity, although it can be corrected by dividing by a sensitivity probability derived from other sources. Furthermore, this approach assumes that the incidence of preclinical cancers is the same as the incidence of clinical cancers at the moment of observation. In the presence of overdiagnosis, this assumption is inaccurate, but if overdiagnosis is not substantial, it may be a useful approximation.

### Maximum likelihood estimation

Using a formal likelihood expression, the duration of the PCDP can be estimated based on the observed prevalence of cancer at screening examinations (i.e., screen-detected cancers), subsequent screens, and the incidence of cancer in the interval between screening examinations (i.e., interval cancers) in relation to the underlying incidence [7,20]. In other words, researchers ask—what PCDP duration is most likely to explain the observed detection rates and incidence rates of interval cancer found in the screening data? This method was applied in 16 of the included studies [7,12,18,20-32]. In this mathematical approach, it is usually assumed that before screening is initiated, the incidence rate of cancer in the population remains constant and that it will remain constant in the absence of screening. Thus, to take account of varying parameters by age, this approach is often performed stratified by age group. This usually involves simply performing separate estimations for several 5-year or 10-year age groups. However, more complex approaches are available. For example, Shen & Zelen [27] and Wu et al. [30] proposed models incorporating the possibility that cancers which would have arisen symptomatically in one age group are diagnosed in a younger age group, due to screening. Hsieh et al. [29] used a parametric model with progression parameters common to all age groups, but with underlying preclinical incidence varying with age. Cong et al. [31] fit 2 models: 1 in which the sensitivity had a linear dependence on age and 1 in which the mean sojourn time had such a dependence.

The first screening examination detects a certain number of cancers that have not yet surfaced clinically. As time passes after the first screening examination, new cancers will develop to become interval cancers, or, in the case of a second examination, will be screen-detected. The incidence of interval cancers in the interval between 2 screening examinations will comprise individuals who had false-negative screening results and newly diagnosed cases. This process will repeat itself during periodic screening examinations. When screening is stopped, the incidence will gradually return to the incidence before the introduction of a cancer screening program [7,20]. With assumed distributions of the relevant observations, a formal statistical likelihood is derived and maximized to estimate the parameters of the preclinical incidence rate, average PCDP duration, and test sensitivity [20].

### Expectation-maximization algorithm

Etzioni & Shen [33] are the only group that described an expectation-maximization algorithm as a tool for obtaining maximum likelihood estimates of the PCDP duration, test sensitivity, and the time of cancer onset. The relationships between these variables are described in the same way as in the maximum likelihood estimation models. An expectation-maximization algorithm is an iterative method to maximize likelihood estimates of the parameters of interest in statistical models, where the model depends on unobserved latent variables. Its advantage is that it simplifies the analytical work that is needed to obtain maximum likelihood estimates from a rather complicated underlying model.

### Regression of observed on expected

Regression of observed on expected, which is a variant of the method of moments, was applied in 6 of the included studies [8,11,34-37]. The method of moments is a method of estimating population parameters in which population moments (e.g., expected values of the variables observed) are expressed as a function of the parameter of interest. Estimates are derived by assuming that the population moments are equal to those observed in the data. This method can sometimes be performed by closed form algebra, and is thus analytically simpler than methods such as maximum likelihood estimation. In regression of observed on expected, the estimation of parameters is performed by letting the observed numbers (for example, the number of screen-detected or interval cancers) equal the expected numbers derived from the underlying assumed model (Supplementary Material 4) plus an error term, using non-linear regression [34,37]. These expected numbers are formulae that include the duration of the PCDP, and may also include sensitivity.

### Bayesian Markov-chain Monte Carlo simulation

Bayesian Markov-chain Monte Carlo simulation methods for estimating the parameters of the PCDP duration were applied in 5 included studies [18,30,38-40]. The posterior joint distribution (usually composed of 3 parameters: the preclinical incidence rate, the average PCDP duration, and test sensitivity) is formed by the prior distributions of the parameters and the likelihood function based on the observed data [43]. The marginal posterior distribution for the parameter of primary interest (i.e., the average duration of the PCDP) can, in theory, be estimated by integrating out other parameters. However, as the marginal posterior distribution is often intractable, Bayesian Markov-chain Monte Carlo simulation (using a sampling algorithm such as the Gibbs sampler), rather than numerical integration, can be used to estimate the parameters. This can be done because after a substantial number of repeated conditional samples, the posterior distribution of the parameter of interest conditional on the other parameters and the data will approximate its marginal distribution.

### Applications in breast and colorectal cancer screening

**Figure 1.**
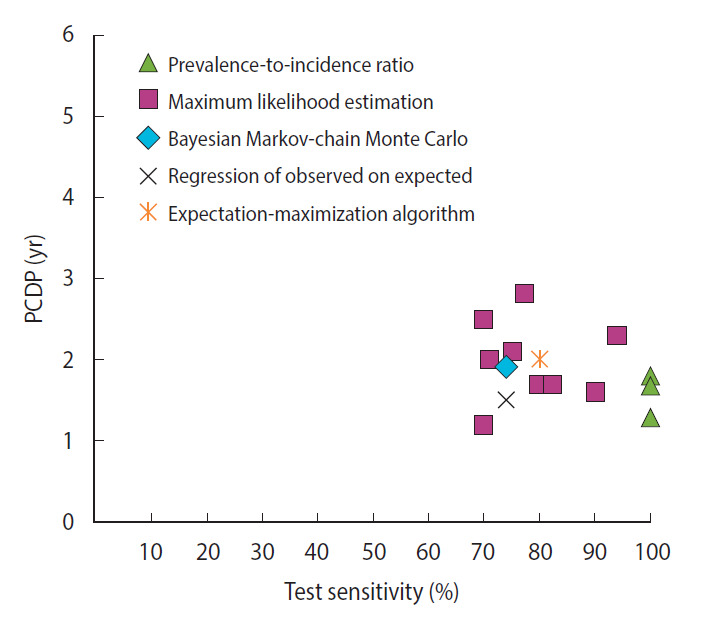
The relation between the average preclinical detectable phase (PCDP) duration and test sensitivity for annual screening for breast cancer comprising mammography and clinical breast examination in women aged 40-64 years (i.e., Health Insurance Plan study, USA, 14 estimates from 13 studies) by mathematical estimation type.

For breast cancer screening, we found 13 studies reporting 14 estimates of the PCDP duration, all using data from the Health Insurance Plan trial, which offered mammography and clinical breast examinations as a screening test [6-8,12-14,20,23,27,30,31,33]. Using the prevalence-to-incidence ratio method, the estimates of the PCDP duration at age 40-64 years were 1.3-1.8 years; test sensitivity was assumed to be 100% (Figure 1) (Supplementary Material 5). The results of the other mathematical estimation approaches (maximum likelihood estimation, Bayesian Markovchain Monte Carlo simulation, regression of observed on expected, and expectation-maximization algorithm) applied to the same data yielded a PCDP duration of 1.2-3.0 years and a test sensitivity ranging from 59% to 90%. Thus, the prevalence-to-incidence method generally led to lower estimates than were obtained using the other methods.

**Figure 2.**
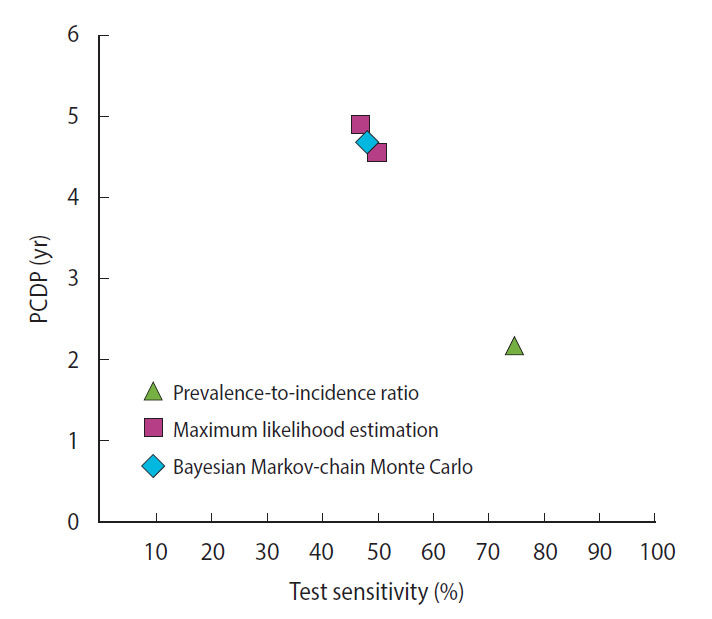
The relation between the average preclinical detectable phase (PCDP) duration and test sensitivity for colorectal cancer screening using guaiac fecal occult blood test (first screen) in men and women aged 45-74 years (i.e., Calvados study, France, 4 estimates from 2 studies) by mathematical estimation type.

For colorectal cancer screening, we identified 2 studies reporting 5 different estimates of the PCDP duration in a guaiac fecal occult blood test screening program in a population aged 45-74 years, all using data from the Calvados study in France (Figure 2) [18,28,32] (Supplementary Material 6). Using the prevalence-to-incidence ratio method, the PCDP duration and test sensitivity were estimated to be 2 years and 75%, respectively. The results of the maximum likelihood estimation and the Bayesian Markov-chain Monte Carlo simulation were a PCDP duration of around 5 years and a test sensitivity of 50% [18,28]. The prevalence-to-incidence estimates of the average PCDP duration were lower than those of the Bayesian Markov-chain Monte Carlo simulation and maximum likelihood estimation.

Other estimates of PCDP duration from studies on breast and colorectal cancer screening programs applied to different screening programs are given in Supplementary Materials 5-8. It should be kept in mind that estimates can only be compared between methods if the methods were used with the same data.

## DISCUSSION

To our knowledge, this is the first systematic review providing a comprehensive overview of mathematical approaches to estimate the duration of the PCDP using prevalence and incidence data alongside screening frequency data, and optionally interval cancer data. We identified 34 approaches that could be classified into 5 distinct mathematical estimation approaches to estimate the duration of the PCDP. When using the same data source, estimates of the PCDP duration varied among the mathematical approaches. Most (n=16, 47%) methods used maximum likelihood estimation. Estimation approaches based on the prevalence-to-incidence ratio generally yielded shorter estimates of the PCDP duration than the other approaches. As the actual duration of the PCDP is unknown, it is complicated to determine which mathematical method is superior.

A comparison of the estimates of the PCDP durations in breast and colorectal cancer screening shed light on the differences resulting from the mathematical approach, assumptions, and data input (Table 4). Prevalence-to-incidence ratio models generally yielded the shortest durations of the PCDP, sometimes but not always because 100% sensitivity was assumed. In these models, sensitivity estimates are nearly 100%, suggesting some estimation instability with this oldest method, despite its simplicity. Estimates of the PCDP durations were longest in models including the regression of observed on expected. A potential explanation could be that these models did not generally take overdiagnosis into account. In addition, the data source is likely to influence estimations of the PCDP duration. The prevalence-to-incidence ratio uses only data from the prevalence screen, and tends to estimate shorter PCDP durations. Estimates that used only the first screen averaged 1.5 years, whereas estimates using subsequent screens and/or interval cancers averaged 2.5 years (Supplementary Material 6). Even when restricted to a single trial, the Health Insurance Plan study of Greater New York, the averages were 1.6 years and 2.0 years, respectively.

Another observation is that 2 studies in the maximum likelihood estimation group based their estimates of the PCDP duration only on interval cancers, estimating longer PCDP durations. This is unexpected, since one would expect estimation from screen-detected cancers to be affected by length bias or overdiagnosis, therefore leading to an overestimation of the PCDP duration, whereas interval cancers would, if anything, be expected to have shorter PCDP durations than screen-detected cancers.

We further found, unsurprisingly, that shorter durations of the PCDP were observed when test sensitivity was assumed to be 100%. This is consistent with the fact that a high detection rate can be modeled as either high test sensitivity or a long duration of the PCDP. Whether the underlying incidence was estimated or observed seemed to have no impact on estimations of the PCDP duration.

It also appeared that models that estimated “nuisance” parameters such as the underlying incidence and test sensitivity were more in line with prior knowledge of already analyzed cancer screening programs than models that constrained some of these parameters from external sources. This might be because the former models better reflect biological complexity or population-specific effects than models with more parameters constrained in advance.

Can we conclude which estimates are best? Not with 100% certainty, but certain remarks can be made. No method is perfect. The major advantage of the prevalence-to-incidence ratio method is its simplicity, while its major disadvantages are the necessity for a number of assumptions and the lack of sound theoretical properties to allow reliable measures of uncertainty. The major advantage of formal statistical methods such as maximum likelihood estimation and Bayesian Markov-chain Monte Carlo simulation is that both give the opportunity to include related parameters (e.g., incidence and test sensitivity) in the model, and yield estimates that take into account the fact that these parameters are also unknown and are estimated with a measure of uncertainty. The disadvantages are the increased complexity and the need for specialized statistics or statistical computing expertise. However, they also allow the calculation of theoretically valid confidence intervals or credibility intervals on the average PCDP duration itself, thus giving more reliable estimates of the uncertainty on this parameter of primary interest. Consequently, we suggest that these are preferable to prevalence-to-incidence ratio models.

The PCDP duration can be used both to design cancer screening programs (e.g., the Swedish breast cancer [44] and the Australian bowel cancer [45] screening programs) and the evaluation of existing programs [46,47]. This review also provides information on the data needed to estimate the duration of the PCDP and can help researchers anticipate this issue when developing cancer screening programs.

Most mathematical approaches included in this review were developed for and applied to breast cancer screening. The current literature is thus less focused on other cancer screening programs such as colorectal and cervical cancer. The latter 2 cancer types, more often than breast cancer, involve the possibility of non-progressive preclinical (and premalignant) states [48]. Only a small number of the included studies took tumor non-progression into account, indicating the need to define the scope of the investigation to develop methods addressing the overdiagnosis of cancer and regression of precancerous lesions, and making it applicable for all cancer types.

## CONCLUSION

The PCDP duration can be estimated by several mathematical estimation approaches that may lead to different estimates. It seems that the simple approaches of the prevalence-to-incidence ratio and regression of observed on expected numbers of cancers have deficiencies that are not shared by more formal statistical approaches, such as maximum likelihood estimation, expectation-maximization algorithm estimation, and Bayesian Markov-chain Monte Carlo simulation. However, we still advise researchers to use multiple estimation approaches, as none of the models is perfect. As in the physical sciences, when one cannot measure something perfectly, a sound approach is to measure it several times by different methods.

## Figures and Tables

**Table 1.** Definitions used in this manuscript

Term	Definition	Reference
PCDP duration	The time interval between the start of the PCDP and the time when the cancer manifests clinically (in the absence of screening); The starting point of the PCDP depends upon characteristics of the screening test, notably its sensitivity	[26]
Sensitivity	The proportion of people with a positive screening test among those who have a cancer in the preclinical detectable phase; Sensitivity is usually considered to be a constant throughout the PCDP, but this is likely to be only approximately true; Intuitively, sensitivity would be lower for smaller tumors earlier in the PCDP and higher for larger tumors later in the PCDP	[1]
Lead time	The duration by which the diagnosis of a cancer is moved forward in time due to detection during a screening examination rather than being detected clinically; In other words, the PCDP duration is the “potential time” a diagnosis of a cancer is moved forward, while lead time is the actual time; Under the assumption of an exponential PCDP distribution, the mean PCDP duration can also be an estimate of the expected lead time for an individual cancer	[20]
Screen-detected cancers	Cancers diagnosed by a screening examination, whereas interval cancers are clinically diagnosed between screening examinations	[12]
Length time bias	Length bias occurs because slowly growing tumors with a favorable prognosis have a longer preclinical detectable phase, and are thus more frequently detected at screening than rapidly growing tumors with an unfavorable prognosis	[1]
Overdiagnosis	The diagnosis of cancer at screening that would never have caused any symptoms or problems during an individual’s lifetime	[41]

PCDP, preclinical detectable phase.

**Table 2.** Overview of the included studies that described mathematical estimation approaches to estimate the duration of the preclinical detectable phase (PCDP) grouped by type of mathematical model and study design

Mathematical approach to estimation	Data source	
	Screened and unscreened populations^1^	Screened population
Prevalence-to-incidence ratio	Hutchinson 1968 (breast) [13]	Launoy 1997 (colorectal) [18]^2^
	Zelen 1969 (breast) [6]	Brenner 2011 (colorectal) [19]
	Shapiro 1974 (breast) [14]	
	Albert 1978 (cervix) [15,16]	
	Louis 1978 (cervix) [17]	
Maximum likelihood estimation	Walter 1983 (breast) [20]	Brookmeyer 1986 (cervix) [24]^3^
	Day 1984 (breast) [7]	Brookmeyer 1987 (cervix) [25]^3^
	Alexander 1989 (breast) [21]	Launoy 1997 (colorectal) [18]^2^
	Shen 2005 (breast) [23]	Straatman 1997 (breast) [26]
		Shen 1999 (breast) [27]
		Pinsky 2001 (colorectal) [28]
		Hsieh 2002 (breast) [29]
		Pinsky 2004 (lung) [22]
		Wu 2005 (breast) [30]^2^
		Cong 2005 (breast) [31]
		Jiang 2016 (breast [12]
		Shen 2019 (breast) [32]
Expectation-maximization algorithm	Etzioni 1997 (breast) [33]	
Regression of observed on expected	Chen 1996 (breast) [34]	Paci 1991 (breast) [8]
	Chen 1997 (breast) [35]	Duffy 1995 (breast) [11]
		Duffy 1997 (breast [36]
		Chen 2000 (breast) [37]
Bayesian Markov-chain Monte Carlo simulation	Myles 2003 (breast) [39]	Launoy 1997 (colorectal) [18]^2^
		Wu 2005 (breast) [30]^2^
		Kim 2015 (breast, lung) [40]
		Shen 2017 (lung) [38]

Values arre presented as author, year (cancer type used as an example).

1 The unscreened population is considered as a control group, and may include the control arm of a randomized controlled trial or a historical control group from the time period before screening.

2 Studies that describe multiple mathematical approaches to estimate the PCDP duration.

3 The study design of these articles was case-control.

**Table 3.** Model assumptions of the included papers by mathematical approach

Study		Model assumptions							
Author, year		Screening round (first and/or subsequent)	Screening data used for estimation (screen-detected and/or interval cancers)	Underlying incidence of cancer (estimated within the model, observed from the control group, observed from registry data or not included)	Assumed distribution(s) of the preclinical detectable phase duration	Lead time modeled (yes, no) and its assumed distribution	Confidence interval or standard error around estimate (yes, no)	Test sensitivity (estimated within the model, assumed 100%, observed from the literature, or not included)	Tumor regression modeled (yes, no), estimates corrected for length time bias and/or overdiagnosis
Prevalence to incidence ratio									
	Hutchinson, 1968 [13]	First	Screen-detected cancer data	Observed from the control group	Constant	Yes, constant	No	Assumed 100%	No, no
	Zelen, 1969 [6]	First	Screen-detected cancer data	Observed from the control group	Exponential	Yes, exponential	No	Assumed 100%	Yes, length time correction
	Shapiro, 1974 [14]	First	Screen-detected and interval cancer data	Observed from the control group (corrected for self-selection)	Exponential	No, NA	No	Assumed 100%	No, no
	Albert, 1978 [15,16]	First	Screen-detected cancer data	Observed from the control group	Exponential, gamma	Yes, exponential	No	Not included	Yes, length time and overdiagnosis correction
	Louis, 1978 [17]								
	Launoy, 1997 [18]^1^	First	Screen-detected and interval cancer	Observed from registry data	Not reported	No, NA	Yes	Estimated within the model	No, no
	Brenner, 2011 [19]	First	Screen-detected cancer data	Observed from registry data	Exponential	No, NA	Yes	Assumed 100%	No, no
Maximum likelihood estimation									
	Walter, 1983 [20]	First and subsequent	Screen-detected and interval cancer data	Observed from the control group	Exponential, log-normal, step function	Yes, NA	Yes	Estimated within the model	No, length time correction
	Day, 1984 [7]	First and subsequent	Screen-detected and interval cancer data	Observed from the control group	Exponential	Yes, exponential	Yes	Estimated within the model	No, length time correction
	Brookmeyer, 1986 [24]	First and subsequent	Screen-detected and interval cancer data	Not included (canceled out the model)	Exponential, piecewise exponential, Weibull, log-normal	No, NA	Yes	Estimated within the model	No, no
	Brookmeyer, 1987 [25]	First and subsequent	Screen-detected and interval cancer data	Not included (canceled out the model)	Exponential	No, NA	Yes	Estimated within the model	Yes, overdiagnosis correction
	Alexander, 1989 [21]	First and subsequent	Screen-detected and interval cancer data	Observed from the control group (corrected for self-selection)	Exponential	Yes, exponential	No	Estimated within the model	No, no
	Launoy, 1997 [18]^1^	First	Screen-detected and interval cancer data	Observed from registry data	Not reported	No, NA	Yes	Estimated within the model	No, no
	Straatman, 1997 [26]	First and subsequent	Screen-detected cancer data	Estimated within the model	Exponential	Yes, exponential	No	Estimated within the model	No, no
	Shen, 1999 [27]	First and subsequent	Screen-detected and interval cancer data	Estimated within the model	Exponential	No, NA	Yes	Estimated within the model	No, no
	Pinsky, 2001 [28]	First	Screen-detected and interval cancer data	Observed from registry data	Exponential, gamma & Weibull	Yes, not reported	Yes	Estimated within the model	No, length time and overdiagnosis correction
	Hsieh, 2002 [29]	First and subsequent	Screen-detected cancer data	Estimated within the model	Weibull and piecewise exponential	No, NA	Yes	Assumed 100%	No, no
	Pinsky, 2004 [22]	First and subsequent	Screen-detected and interval cancer data	Estimated within the model	Exponential, Weibull	Yes, not reported	Yes	Estimated within the model	No, overdiagnosis correction
	Shen, 2005 [23]	First and subsequent	Screen-detected and interval cancer data	Observed from the control group	Piecewise-constant	No, NA	No	Estimated within the model	No, no
	Wu, 2005 [30]1	First and subsequent	Screen-detected and interval cancer data	Estimated within the model	Log-logistic	No, NA	No	Estimated within the model	No, no
	Cong, 2005 [31]	First and subsequent	Screen-detected and interval cancer data	Estimated within the model	Exponential	No, NA	Yes	Estimated within the model	No, no
	Jiang, 2016 [12]	First and subsequent	Screen-detected and interval cancer data	Not included (assumed constant)	Exponential	No, NA	Yes	Estimated within the model	No, no
	Shen, 2019 [32]	First and subsequent	Screen-detected and interval cancer data	Estimated within the model	Exponential	No, NA	Yes	Observed from literature	Yes, no
Expectation-maximization algorithm									
	Etzioni, 1997 [33]	First and subsequent	Screen-detected and interval cancer data	Observed from the control group	NA	No, NA	No	Estimated within the model	No, no
Regression of observed on expected									
	Paci, 1991 [8]	First and subsequent	Interval cancer data	Estimated within the model	Exponential	Yes, exponential	Yes	Estimated within the model	No, no
	Duffy, 1995 [11]	First and subsequent	Interval cancer data	Estimated within the model	Exponential	No, NA	Yes	Assumed 100%	No, no
	Chen, 1996 [34]	First and subsequent	Screen-detected and interval cancer data	Observed from the control group	Exponential	No, NA	Yes	Estimated within the model	No, no
	Chen, 1997 [35]	First and subsequent	Screen-detected and interval cancer data	Observed from the control group	Exponential	No, NA	Yes	Assumed 100% or observed from the literature	No, no
	Duffy, 1997 [36]	First and subsequent	Screen-detected and interval cancer data	Estimated within the model	Exponential	No, NA	Yes	Estimated within the model	No, no
	Chen, 2000 [37]	First and subsequent	Screen-detected cancer data	Estimated within the model	Not reported	No, NA	Yes	Assumed 100% or estimated within the model	No, no
Bayesian Markov-chain Monte Carlo simulation									
	Launoy, 1997 [18]^1^	First	Screen-detected and interval cancer data	Observed from registry data	Not reported	No, NA	Yes	Estimated within the model	No, no
	Myles, 2003 [39]	First and subsequent	Screen-detected and interval cancer data	Observed from the control group	Poisson	No, NA	Yes	Estimated within the model	No, no
	Wu, 2005 [30]1	First and subsequent	Screen-detected and interval cancer data	Estimated within the model	Non-parametric	No, NA	Yes	Estimated within the model	No, no
	Kim, 2015 [40]	First and subsequent	Screen-detected interval cancer data	Estimated within the model	Log-logistic	No, NA	Yes	Estimated within the model	No, no
	Shen, 2017 [38]	First and subsequent	Screen-detected and interval cancer data	Estimated within the model	Exponential	No, NA	Yes	Assumed 100% or estimated within the model	Yes, overdiagnosis correction

correctionNA, not available.

1 Articles that described multiple methods to estimate the preclinical detectable phase duration.

**Table 4.** Summary of the method-specific findings

Factors	Summary of findings
Type of mathematical estimation approach	Prevalence-to-incidence ratio models tend to give shorter estimates, and regression of observed on expected tends to give longer estimates of the PCDP duration and higher test sensitivities than other mathematical estimation approaches
Data used	The use of only prevalence screening data tends to give shorter estimates, whereas the use of only interval cancer data tends to give longer estimates of the PCDP durations than using both
Test sensitivity	Shorter durations of the PCDP are observed if 100% test sensitivity is assumed (these studies were predominated by the Health Insurance Plan study data with a younger population and 1960s film technology mammography) than when test sensitivity was estimated within the model
Underlying incidence	No impact on the PCDP duration

PCDP, preclinical detectable phase.

## SUPPLEMENTARY MATERIALS

Supplementary Material 1. Embase and PubMed search strategy with used search terms.

Supplementary Material 2 Study selection for inclusion in the review.

Supplementary Material 3. Model assumptions of the included papers by mathematical approach.

Supplementary Material 4. Basic algebraic development of the mathematical approaches

Supplementary Material 5. Estimates of the preclinical detectable phase duration for breast cancer when screening included annual mammography and clinical breast examination (Health Insurance Plan study, Canadian National Breast Screening Studies and Edinburgh Randomized Trial of Breast Cancer Screening) with a description of the mathematical approach to estimation and model assumptions.

Supplementary Material 6. Estimates of the preclinical detectable phase duration for colorectal cancer in guaiac fecal occult blood test screening (Calvados study, France) with a description of the mathematical approach to estimation and model assumptions.

Supplementary Material 7. Estimates of the preclinical detectable phase duration for breast cancer when screening included mammography only (Two-County trial, Nijmegen Screening Program, Florence District Screening Program and Ontario Breast Screening Program) with a description of the mathematical approach to estimation and model assumptions.

Supplementary Material 8. Estimates of the preclinical detectable phase duration for colorectal cancer in colonoscopy screening (German screening program, Germany) with a description of the mathematical approach to estimation and model assumptions.
