# Supplementary material for: Quantifying the duration of the preclinical detectable phase in cancer screening: a systematic review
Source: Epidemiol Health. 2022 Jan 3;44:e2022008. doi: 10.4178/epih.e2022008 (PMC9117108; doi:10.4178/epih.e2022008)
Supplement: Supplementary Material 1. — Embase and PubMed search strategy with used search terms. [file epih-44-e2022008-suppl1.doc]

**Supplementary Material for ‘Quantifying the duration of the preclinical detectable phase in cancer screening: a systematic review’ by Geurts et al.**

**Supplementary Material 1.** Embase and PubMed search strategy with used search terms.

| **Database** | **Part of the search** | **Search terms (Mesh and free text)** |
| --- | --- | --- |
| **Embase** | **Cancer** | "Neoplasms"[Mesh] OR cancer.ti,ab. OR cancers.ti,ab. OR carcinoma.ti,ab. OR neoplasm.ti,ab. OR neoplasms.ti,ab. OR .ti,ab. OR s.ti,ab. OR tumour.ti,ab. OR tumours.ti,ab. OR neoplasia.ti,ab. |
|  | **Screening** | "Mass Screening"[Mesh] OR screening.ti,ab. OR screenings.ti,ab. OR screen.ti,ab. OR screens.ti,ab. OR screened.ti,ab. OR screen-detected.ti,ab. OR screen detected.ti,ab. OR "Early Detection of Cancer"[Mesh] OR early Detection.ti,ab. OR early diagnosis.ti,ab. |
|  | **Preclinical detectable phase (sojourn time)/lead time*)** | lead time.ti,ab. OR lead-time.ti,ab. OR sojourn time.ti,ab. OR sojourn-time.ti,ab. OR Pre-clinical disease state.ti,ab. OR preclinical disease state.ti,ab. OR Pre-clinical detectable phase.ti,ab. OR preclinical detectable phase.ti,ab. OR detectable preclinical phase.ti,ab. OR preclinical duration.ti,ab. OR preclinical detectable disease state.ti,ab. |
| **PubMed** | **Cancer** | "Neoplasms"[Mesh] OR cancer[tiab] OR cancers[tiab] OR carcinoma[tiab] OR neoplasm[tiab] OR neoplasms[tiab] OR [tiab] OR s[tiab] OR tumour[tiab] OR tumours[tiab] OR neoplasia[tiab] |
|  | **Screening** | "Mass Screening"[Mesh] OR screening[tiab] OR screenings[tiab] OR screen[tiab] OR screens[tiab] OR screened[tiab] OR screen-detected[tiab] OR screen detected[tiab] OR "Early Detection of Cancer"[Mesh] OR early Detection[tiab] OR early diagnosis[tiab] |
|  | **Preclinical detectable phase (sojourn time)/lead time*)** | lead time[tiab] OR lead-time[tiab] OR sojourn time[tiab] OR sojourn-time[tiab] OR Pre-clinical disease state[tiab] OR preclinical disease state[tiab] OR Pre-clinical detectable phase[tiab] OR preclinical detectable phase[tiab] OR detectable preclinical phase[tiab] OR preclinical duration[tiab] OR preclinical detectable disease state[tiab] |

* Lead time was included in the search strategy as we found out that this term is sometimes mixed-up with the preclinical detectable phase duration.
