# Supplementary material for: Quantifying the duration of the preclinical detectable phase in cancer screening: a systematic review
Source: Epidemiol Health. 2022 Jan 3;44:e2022008. doi: 10.4178/epih.e2022008 (PMC9117108; doi:10.4178/epih.e2022008)
Supplement: Supplementary Material 2 — Study selection for inclusion in the review. [file epih-44-e2022008-suppl2.doc]

Records identified through the Pubmed database (n = 532)

Records identified through the Embase database (n = 647)

Full-text articles assessed for eligibility (n = 255)

Records excluded (n = 434)

Studies included in the review (n = 33)

Records excluded based on the following criteria (n = 222):

No full text available: 8

No primary research: 43

Only lead time correction/no lead time estimation: 74

Cancer follow-up: 5

Tumour growth/microsimulation: 16

Overdiagnosis: 3

Application only: 50

Biomarker: 17

Other: 6

Records after removal of duplicates (n = 689)

Records screened for relevance based on title and abstract (n=689)

**Supplementary Material 2.** Study selection for inclusion in the review
