# Supplementary material for: Quantifying the duration of the preclinical detectable phase in cancer screening: a systematic review
Source: Epidemiol Health. 2022 Jan 3;44:e2022008. doi: 10.4178/epih.e2022008 (PMC9117108; doi:10.4178/epih.e2022008)
Supplement: Supplementary Material 3. — Model assumptions of the included papers by mathematical approach. [file epih-44-e2022008-suppl3.doc]

**Supplementary Material 3. Model assumptions of the included papers by mathematical approach.**

| **Article (Author, years)** | **Screening round (first and/or subsequent)** | **Test sensitivity** | **Screening data used for estimation** |
| --- | --- | --- | --- |
|
|
| **Prevalence to incidence ratio** | | | |
| Hutchinson, 1968  [1] | First | Assumed 100% | Screen-detected cancer data |
| Zelen, 1969  [2] | First | Assumed 100% | Screen-detected cancer data |
| Shapiro, 1974  [3] | First | Assumed 100% | Screen-detected and interval cancer data |
| Albert I-III, 1978  [4-6] | First | Not included | Screen-detected cancer data |
| Launoy, 1997^*^  [7] | First | Estimated within the model | Screen-detected and interval cancer |
| Brenner, 2011  [8] | First | Assumed 100% | Screen-detected cancer data |
| **Maximum likelihood estimation** | | | |
| Walter, 1983  [9] | First and subsequent | Estimated within the model | Screen-detected and interval cancer data |
| Day, 1984  [10] | First and subsequent | Estimated within the model | Screen-detected and interval cancer data |
| Brookmeyer, 1986  [11] | First and subsequent | Estimated within the model | Screen-detected and interval cancer data |
| Brookmeyer, 1987  [12] | First and subsequent | Estimated within the model | Screen-detected and interval cancer data |
| Alexander, 1989  [13] | First and subsequent | Estimated within the model | Screen-detected and interval cancer data |
| Launoy, 1997^*^  [7] | First | Estimated within the model | Screen-detected and interval cancer data |
| Straatman, 1997  [14] | First and subsequent | Estimated within the model | Screen-detected cancer data |
| Shen, 1999  [15] | First and subsequent | Estimated within the model | Screen-detected and interval cancer data |
| Pinsky, 2001  [16] | First | Estimated within the model | Screen-detected and interval cancer data |
| Hsieh, 2002  [17] | First and subsequent | Assumed 100% | Screen-detected cancer data |
| Pinsky, 2004  [18] | First and subsequent | Estimated within the model | Screen-detected and interval cancer data |
| Shen, 2005  [19] | First and subsequent | Estimated within the model | Screen-detected and interval cancer data |
| Wu, 2005^*^  [20] | First and subsequent | Estimated within the model | Screen-detected and interval cancer data |
| Cong, 2005  [21] | First and subsequent | Estimated within the model | Screen-detected and interval cancer data |
| Jiang, 2016  [22] | First and subsequent | Estimated within the model | Screen-detected and interval cancer data |
| Shen, 2019  [23] | First and subsequent | Observed from literature | Screen-detected and interval cancer data |
| **Expectation-maximization algorithm** | | | |
| Etzioni, 1997  [24] | First and subsequent | Estimated within the model | Screen-detected and interval cancer data |
| **Regression of observed on expected** | | | |
| Paci, 1991  [25] | First and subsequent | Estimated within the model | Interval cancer data |
| Duffy, 1995  [26] | First and subsequent | Assumed 100% | Interval cancer data |
| Chen, 1996  [27] | First and subsequent | Estimated within the model | Screen-detected and interval cancer data |
| Chen, 1997  [28] | First and subsequent | Assumed 100% or observed from literature | Screen-detected and interval cancer data |
| Duffy, 1997  [29] | First and subsequent | Estimated within the model | Screen-detected and interval cancer data |
| Chen, 2000  [30] | First and subsequent | Assumed 100% or estimated within the model | Screen-detected cancer data |
| **Bayesian Markov Chain Monte Carlo simulation** | | | |
| Launoy, 1997^*^  [7] | First | Estimated within the model | Screen-detected and interval cancer data |
| Myles, 2003  [31] | First and subsequent | Estimated within the model | Screen-detected and interval cancer data |
| Wu, 2005^*^  [20] | First and subsequent | Estimated within the model | Screen-detected and interval cancer data |
| Kim, 2015  [32] | First and subsequent | Estimated within the model | Screen-detected interval cancer data |
| Shen, 2017  [33] | First and subsequent | Assumed 100% or estimated within the model | Screen-detected and interval cancer data |
