# Supplementary material for: Quantifying the duration of the preclinical detectable phase in cancer screening: a systematic review
Source: Epidemiol Health. 2022 Jan 3;44:e2022008. doi: 10.4178/epih.e2022008 (PMC9117108; doi:10.4178/epih.e2022008)
Supplement: Supplementary Material 4. — Basic algebraic development of the mathematical approaches [file epih-44-e2022008-suppl4.doc]

**Supplementary Material 4**

**Basic algebraic development of the mathematical approaches**

**1. Prevalence to incidence ratio**

The number of cancers at a specific point in time (i.e. the prevalence) depends on the rate with which the new preclinical disease develops in the population and enters the detectable phase (i.e. the underlying incidence) and the length of time cancers spend in this phase before clinical symptoms arise (i.e. the duration of the preclinical detectable phase)  [1]. If the incidence rate is I, the duration of the preclinical detectable phase is D and the prevalence rate is P, this relation is written as:

P = D x I

and D can be estimated as

D = P/I

The above assumes 100% sensitivity. If external estimates or further data are available to estimate the sensitivity (S), we can estimate D as

D = P/(I x S)

An implicit assumption is that these quantities do not depend on calendar time  [2, 34]. In other words, if the number of prevalent preclinical cancers in a given population remains constant over time, this means that new cases enter the preclinical detectable phase at the same rate as preclinical cancers are becoming clinical. So, the incidence of preclinical cancers must be the same as the incidence of clinical cancers at the moment of observation.

**Example 1: breast cancer screening program in Nijmegen, the Netherlands  [35, 36]**

**Input:** This method uses the screen-detected cancer data of the first screening round for the prevalence rate (P). The incidence rate (I) was either based on the incidence rate found in the control group or based on an incidence rate collected from a registry in the time period before the existence of the screening program or from a similar region. Test sensitivity is mostly not taken into account and assumed to be perfect, i.e. 100%.

**P=** Screen-detected cancer data: number of cancers detected during a screening round per women screened in that round. E.g. Nijmegen (1975-1988, age 50-69 years): 260/65,404 = 4.0 per 1000 women screened.

**I=** Incidence rate in the control group. E.g. Arnhem (1975-1982, age 50-69 years): 208/11,0523 = 1.9 per 1000 women-years.

**D=** 4.0/1.9= 2.1 years, the estimate of preclinical detectable phase duration.

**Maximum likelihood estimation**

The incidence of breast cancer at time t, I(t), and the prevalence of breast cancer at a screening examination n, P(n), can be seen as a function of the underlying incidence rate I, the false-negative rate β, and the preclinical detectable phase of the disease PCDP:

I(t) = f (I, β, PCDP, t)

P(t) = g (I, β, PCDP, n)

With suitable distributions assumed for I(t) and P(t), the probability of the observed values of I and P can be calculated, and this can be converted to a likelihood or log-likelihood. We then find the parameter values of β, PCDP and so on which give the maximum values for the likelihood or log-likelihood.

**Example 2: breast cancer screening program in Nijmegen, the Netherlands  [35]**

**Input:** This method uses the screen-detected cancers (separate for first and subsequent screens) and/or interval cancer data (per year). E.g. Nijmegen (1975-1988, age 50-69 years):

- Total screen-detected cancers: 260
- Total interval cancers: 130
- Interval cancer year 1: 38
- Total first screening (prevalent): 12,317
- Total screen-detected cancers first screening: 69
- Total interval cancers first screening: 18
- Total subsequent screening (incident): 53,087
- Total screen-detected cancers subsequent screening: 191
- Total interval cancer subsequent screening: 112
- Screenings interval (t): 2 years
- Sensitivity: 87%

**Estimation:**

Two of the three parameters (underlying incidence, preclinical detectable phase duration and sensitivity) can be maximized using maximum likelihood estimation if the third is derived from an independent sources. Four steps have to be followed:

1. Set ranges for parameters:

- - - *λ_1_* (underlying incidence of preclinical disease): 0.0010-0.0049
    - *λ_2_*  (progression rate to symptomatic disease): 0.20-0.59

2. Generate expected probabilities P, I, C, based on *λ_1_* and *λ_2_*

- - - P (expected proportion of cancers detected at first screen)=
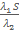

    - I (expected proportion of cancers detected at subsequent screen) =
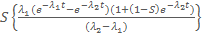

    - C (expected proportion of screened negative subjects having a clinical symptomatic interval cancer before the next screen
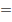


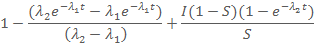


3. Generate log-likelihoods (separate for first and subsequent screens) of empirical values (i.e. the observed screening data, see input above), given the expected probabilities

4. Perform maximum-likelihood estimation in order to get point estimates for *λ_1_*and *λ_2_*.

**Output**:

1/*λ_2_*= 1/0.40= 2.5 years, the estimate of preclinical detectable phase duration.

**3. Expectation-maximization algorithm**

The likelihood above can be computationally complex and convergence to an estimate may be facilitated or accelerated by use of the EM-algorithm. The EM iteration alternates between the performance of the expectation (E) step and the maximization (M) step. The E step derives the expectation of the log-likelihood evaluated using the current estimate for the latent variables. The M step estimates the model parameters (in this case the average preclinical detectable phase duration and sensitivity) by maximizing the expected log-likelihood found on the E step. These parameter estimates are then used to re-estimate the distribution of the latent variables in the next E step. This process is repeated until convergence, i.e. until the (n+1)th estimates are indistinguishable from the nth to a certain number of decimal places.

**Example 3: Health Insurance Plan (HIP) breast cancer screening trial, USA**

The expectation-maximization (EM) algorithm described in the study of Etzioni and Shen  [24] can be used to estimate the preclinical detectable phase duration without making parametric assumptions about the form of its distribution. The absence of a distributional assumption implies a complex likelihood, for which the EM algorithm can be helpful. The EM algorithm can be thought of simply as a way of simultaneously estimating two sets of parameters from a complicated likelihood: it calculates the expected values of set 1 conditional on the values of set 2, then maximizes the likelihood conditional on the expected values of set 1 to obtain maximum likelihood estimates of set 2. Then the expected values for set 1 are recalculated conditional on the new values of set 2, and the process is repeated until convergence.

Etzioni and Shen  [24] re-express the problem as a series of discrete rather than continuous time points, where time j (= 0,1,2,…) is the time of onset of the preclinical disease phase and k (=0,1,2,…) is the duration of the preclinical detectable phase. Every subject in the data set can have different values of j and k. For a given person i (i = 1,2,3, …, n, where n is the total number of individuals in the dataset), they define I_jk_^i^ as 1 if for person I, time of onset is j and sojourn time is k. The M-step of the algorithm is the estimation of the expected value of I_jk_^i^ for each individual I as the probability that I_jk_^i^ is 1, weighted by the implied probabilities of false negative and true positive screening tests divided by the weighted sum of probabilities of all possible combinations of i and j for that individual. The E-step is then to obtain maximum likelihoods of those probabilities of i and j conditional on these expected values of I_jk_^i^.

**Input:** The EM algorithm was applied to the HIP breast cancer screening trial in which 60,000 women between the ages of 40 and 64 were enrolled and annually screened by mammography. Ten-year follow-up data were available for 27,632 women in the screening group with a known disease state (alive, dead without breast cancer, breast cancer case). Of these, there were 611 breast cancers. Because of the heavy computing burden, the authors used all the cancers but only 10% of the non-cancers for estimation.

**Estimation:**

Because of the complication of simultaneously estimating the probabilities of the unobserved time of onset of the preclinical screen detectable period, the preclinical detectable phase duration and the sensitivity, the authors held the last parameter constant at a series of values and estimated the other parameters (which in turn yielded maximum likelihood estimates of the mean preclinical detectable phase duration) and chose among those constant values the sensitivity which gave the highest likelihood value after convergence.

**Output:**

Probably due to the rather small number of cancers, the data were consistent with a range of sensitivities, from around 0.3 to 0.7, and mean preclinical detectable phase durations from 1 to 4 years.

**4. Regression of observed on expected**

A three-state Markov model can be applied to depict the progression process of cancer from the states of free from cancer (state 0), to preclinical disease (state 1), and clinical disease (state 2). Cancers detected at a screening examination were those in the preclinical detectable phase (PCDP) and interval cancers in the clinical phase (CP)  [27, 30]. Let the underlying incidence of preclinical disease and the rate of disease progression from preclinical to clinical phase be denoted below by *λ_1_* and *λ_2_*, respectively. The intensity matrix of the three-state model is thus


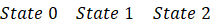


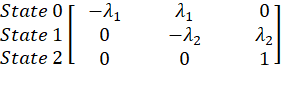


and the corresponding transition probability matrix for the interval between screening examinations in year *t*


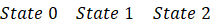


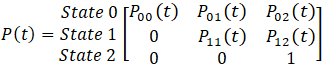


The following formulae follow from the Markov assumptions.


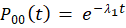
,


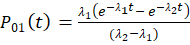
,


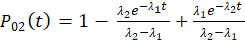
,


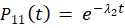
,


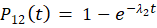
,

The probabilities of observing preclinical cancers and subjects free from cancer in the prevalent screening round are thus


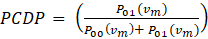
,


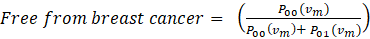
 (A)

where *v_m_* is age at prevalent screening exam. The probabilities of observing clinical disease or preclinical disease, and subjects free from cancer in the subsequent round are thus

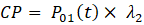


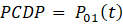


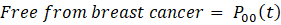
 (B)

where *t* is the interval between screening examination in years. Estimation of parameters can be performed from the above series of equations by letting the observed numbers equal the expected plus an error term for each mode of detection using non-linear regression  [27, 30]. For the estimation of test sensitivity, the probabilities in formula (A) and (B) are extended as follows:


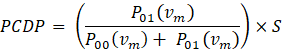


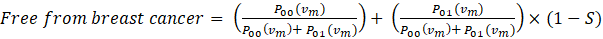
 (C) and


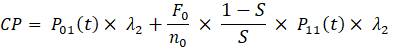


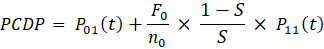


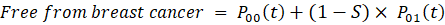
 (D)

where *F_0_* represents the total number of cancers detected in the previous screening round and *n_0_* represents the number of attendees at previous exams  [30]. Considering the model taking into account the heterogeneity in transition rates, a regression model was adopted as follows:

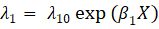


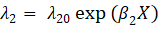


where *X* is a vector of dummy variables corresponding to each period with the effect on underlying cancer incidence rate and rate of progression through regression coefficients *β_1_* and *β_2_*, respectively, and *λ_10_* and *λ_20_* representing the baseline transition rates. The heterogeneity in sensitivity (S) of each period was modeled using a logistic form


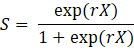


The parameters were estimated by non-linear regression, with the expected numbers of cancer at first and subsequent screening exams, and numbers of interval cancers based on the above formulae as the regression predictor and the observed numbers as the dependent variable  [27].

**Example 4: bowel cancer screening program in Calvados, France  [7]**

Paci and Duffy  [25] give the expected number of interval cancers in a given year after screening as:


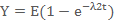


where t is the year since screening, E is the expected number of cancers in that year if screening had not taken place and λ_2_ is the transition rate from asymptomatic screen-detectable to symptomatic cancer, that is λ_2_=1/PCDP.

**Input:**

Launoy et al.  [7] reported interval cancer incidence following fecal occult blood test screening in 69,810 individuals in the first, second, and third year since screen in Calvados, France, see table:

| Year since screen | Interval cancers | Person-years | Incidence per 100,000 in the absence of screening | Expected number of cancers in the absence of screening |
| --- | --- | --- | --- | --- |
| 1 | 44 | 68,916 | 83 | 57.2 |
| 2 | 29 | 63,735 | 83 | 52.9 |
| 3 | 22 | 40,120 | 83 | 33.3 |

They estimate incidence in the absence of screening as 83 per 100,000 per year, from rates observed in the years immediately preceding the screening program. The expected numbers of cancers in the absence of screening is therefore estimated as the person-years times 0.00083.

**Estimation:**

If we input into STATA version 16 the years since screen as t = 1,2,3, interval cancers as can = 44,29,22, and expected numbers as ei = 57.2,52.9,33.3, we can use the non-linear regression option in STATA:

nl(can=ei*(1-exp(-1*{lam=0.3}*t)))

**Output**:

1/ *λ_2_*= 1/0.76= 1.32 years, the estimate of preclinical detectable phase duration.

**Bayesian Markov Chain Monte Carlo estimation**

This uses essentially the same probability formulae as in maximum likelihood above, and if a Markov process model is assumed for disease progression, the same probabilities as in regression of observed on expected above. However, in the Bayesian Markov Chain Monte Carlo approach, the parameters are treated as random variables, each with its prior distribution. From the assumed distributional forms and the probability formulae, condition posterior distributions of the parameters including the preclinical detectable phase duration are derived and sampled from. The sampling is repeated until sufficient numbers of samples give precise estimates of the mean and variation of the parameters. As noted in the main text, the convergence to the marginal posterior distribution of the parameter of interest avoids the necessity of complex numerical integration of the likelihood function.

**Example 5: breast cancer screening in women with a family history in Kopparberg, Sweden and Manchester, UK  [31]**

The Bayesian Markov Chain Monte Carlo approach is a Bayesian approach which treats the parameter of interest as a random variable. The process repeatedly samples from the distribution of that random variable conditional on the previous value and on the data. Under certain conditions, the conditional distribution converges to the unconditional posterior distribution of the parameter of interest. Myles et al.  [31] used the method to estimate sensitivity and the preclinical detectable phase duration simultaneously from the subset of screened women with a family history of breast cancer in the Swedish Two County Trial of mammographic screening.

Myles et al.  [31] took data from two screening services in women with a family history of breast cancer. From each, data were available on the number of prevalent (first) screens and corresponding cancers diagnosed, numbers of incident (subsequent) screens and corresponding cancers, and numbers of interval cancers.

**Input:**

The data used for estimation from the Swedish Two County Trial were as follows:

- Number of persons screened: 3226
- Total prevalent screen cancers: 15
- Total interval cancers: 15
- Total incident screen cancers: 14

The numbers from Manchester were:

- Number of persons screened: 2998
- Total prevalent screen cancers: 15
- Total interval cancers: 9
- Total incident screen cancers: 26

**Estimation:**

They modelled numbers of screen detected cancers as binomial with probabilities depending on the screening sensitivity, underlying incidence of preclinical screen-detectable cancer, transition rate from preclinical to clinical disease (inverse of preclinical detectable phase duration) and whether the screen was prevalent or incident, and numbers of interval cancers as Poisson distributed with probabilities depending on screening sensitivity, underlying incidence of preclinical cancer, transition rate to clinical disease and time since screen. They assumed a common transition rate for the two centers, but estimated separate sensitivities, and constrained the underlying incidence to separate values based on incidence rates local to the two screening services. They placed vague priors on the parameters to be estimated, in order that the resulting estimates would approximate maximum likelihood estimates.

The computational resampling from conditional posterior distributions of the unknown parameters was obtained by Gibbs sampling within the BUGS program. The full details of assumptions and computing code are given by Myles et al.  [31].

**Output:**

After convergence, the method yielded an estimate of 1.80 years (95% credible interval 1.33-2.45) and sensitivities of 94.6% (95% credible interval 88.5%-99.3%) for Sweden and 98.1% (70.9%-99.3%) for Manchester.
