# Supplementary material for: Quantifying the duration of the preclinical detectable phase in cancer screening: a systematic review
Source: Epidemiol Health. 2022 Jan 3;44:e2022008. doi: 10.4178/epih.e2022008 (PMC9117108; doi:10.4178/epih.e2022008)
Supplement: Supplementary Material 5. — Estimates of the preclinical detectable phase duration for breast cancer when screening included annual mammography and clinical breast examination (Health Insurance Plan study, Canadian National Breast Screening Studies and Edinburgh Randomized Trial of Breast Cancer Screening) with a description of the mathematical approach to estimation and model assumptions. [file epih-44-e2022008-suppl5.doc]

**Supplementary Material 5.** Estimates of the preclinical detectable phase duration for breast cancer when screening included annual mammography and clinical breast examination (Health Insurance Plan study, Canadian National Breast Screening Studies and Edinburgh Randomized Trial of Breast Cancer Screening) with a description of the mathematical approach to estimation and model assumptions.

| **Author, year** | **Data used** | **Type of mathematical model** | **Age range**  **(year)** | **Overall preclinical detectable phase duration in years (standard error or 95% confidence interval)** | **Test sensitivity in percentage (standard error or 95% confidence interval)** |
| --- | --- | --- | --- | --- | --- |
| **Health Insurance Plan study, USA** | | | | | |
| Hutchison, 1968  [1] | Screen-detected cancer data, incidence observed from control group | Prevalence to incidence ratio | 40-64 | 1.7 | Assumed 100 |
| Zelen, 1969  [2] | Screen-detected cancer data, incidence observed from control group | Prevalence to incidence ratio | 40-64 | 1.8 | Assumed 100 |
| Shapiro, 1974  [3] | Screen-detected cancer data, incidence observed from control group | Prevalence to incidence ratio | 40-64 | 1.3 | Assumed 100 |
| Walter, 1983  [9] and Day, 1984  [10] | Screen-detected and interval cancer data, incidence observed from control group | Maximum likelihood estimation | 40-64 | 1.7 (1.1 ― 2.9) | 82 (51 ― 100) |
| Paci, 1991  [25] | Interval cancer data, incidence estimated within the model | Regression of observed on expected | 40-64 | 1.5 (1.1 ― 2.2) | 74 |
| Etzioni, 1997  [24] | Screen-detected and interval cancer data, incidence observed from control group | Expectation-maximization algorithm | 40-64 | 2.0 | 80 |
| Shen, 1999  [15] | Screen-detected and interval cancer data, incidence estimated within the model | Maximum likelihood estimation | 40-64 | 2.5 (standard error: 1.2) | 70 (standard error: 20) |
| Wu, 2005  [20] | Screen-detected and interval cancer data, incidence estimated within the model | Maximum likelihood estimation | 40-64 | 1.8 | Not reported |
| Wu, 2005  [20] | Screen-detected and interval cancer data, incidence estimated within the model | Bayesian Markov Chain Monte Carlo | 40-64 | 1.9 | 74 |
| Cong, 2005  [21] | Screen-detected and interval cancer data, incidence estimated within the model | Maximum likelihood estimation, assumed constant sojourn time and sensitivity | 40-64 | 2.1 (standard error: 0.6) | 75 (standard error:15) |
| Cong, 2005  [21] | Screen-detected and interval cancer data, incidence estimated within the model | Maximum likelihood estimation, sensitivity varying with age | 40-64 | 2.6 (standard error: 0.5) | Varies with age |
| Cong, 2005  [21] | Screen-detected and interval cancer data, incidence estimated within the model | Maximum likelihood estimation, sojourn time varying with age | 40-64 | 3.0 at age 40 | 59 (standard error: 15) |
| Shen, 2005  [19] | Screen-detected and interval cancer data, incidence observed from control group | Maximum likelihood estimation | 40-64 | 2.0 (standard error: 0.04) | 71 (standard error: 13) |
| Jiang, 2016  [22] | Screen-detected and interval cancer data, incidence not included | Maximum likelihood estimation | 40-64 | 2.3 | 94 |
| Shen, 2019  [23] | Screen-detected and interval cancer data, incidence estimated within the model | Maximum likelihood estimation | 40-64 | 1.2 1.7 1.6 | 70 80 90 |
| **Canadian National Breast Screening Studies, Canada** | | | | | |
| Shen, 1999  [15] | Screen-detected and interval cancer data, incidence estimated within the model | Maximum likelihood estimation | 40-49  50-59 | 2.1 (standard deviation: 1.5)  3.8 (standard deviation: 1.3) | 86 (standard deviation: 22)  78 (standard deviation: 13) |
| Cong, 2005  [21] | Screen-detected and interval cancer data, incidence estimated within the model | Maximum likelihood estimation, assumed constant sojourn time and sensitivity | 40-59 | 2.7 (standard error: 0.5) | 82 (10) |
| Cong, 2005  [21] | Screen-detected and interval cancer data, incidence estimated within the model | Maximum likelihood estimation, sensitivity varying with age | 40-59 | 2.4 (standard error: 1.2) | Varies with age, average 90 |
| Cong, 2005  [21] | Screen-detected and interval cancer data, incidence estimated within the model | Maximum likelihood estimation, sojourn time varying with age | 40-59 | 2.9 varies with age | 80 (20) |
| Shen, 2005  [19] | Screen-detected and interval cancer data, incidence observed from control group | Maximum likelihood estimation | 40-49  50-59 | 2  3.3 | 84 (standard error: 16)  74 (standard error: 8) |
| Shen, 2019  [23] | Screen-detected and interval cancer data, incidence estimated within the model | Maximum likelihood estimation | 40-49 40-49 40-49 50-59 50-59 50-59 | 3.4 3.6 2.7 2.3 2.4 3.4 | 70 80 90 70 80 90 |
| **Edinburgh Randomized Trial of Breast Cancer Screening, UK** | | | | | |
| Alexander, 1989  [13] | Screen-detected and interval cancer data, incidence observed from control group | Maximum likelihood estimation | 45-64 | 5.0 | 63 |
