# Supplementary material for: Quantifying the duration of the preclinical detectable phase in cancer screening: a systematic review
Source: Epidemiol Health. 2022 Jan 3;44:e2022008. doi: 10.4178/epih.e2022008 (PMC9117108; doi:10.4178/epih.e2022008)
Supplement: Supplementary Material 6. — Estimates of the preclinical detectable phase duration for colorectal cancer in guaiac fecal occult blood test screening (Calvados study, France) with a description of the mathematical approach to estimation and model assumptions. [file epih-44-e2022008-suppl6.doc]

**Supplementary Material 6.** Estimates of the preclinical detectable phase duration for colorectal cancer in guaiac fecal occult blood test screening (Calvados study, France) with a description of the mathematical approach to estimation and model assumptions.

| **Author, year** | **Data used** | **Type of mathematical model** | **Age range**  **(years)** | **Overall preclinical detectable phase duration in years (95% confidence interval)** | **Test sensitivity in percentage (95% confidence interval)** |
| --- | --- | --- | --- | --- | --- |
| Launoy, 1997  [7] | Screen-detected and interval cancer data, incidence observed from registry data | Bayesian Markov Chain Monte Carlo estimation | 45-74 | 4.7 (3.1 – 8.4) | 48 (30 – 66) |
| Launoy 1997  [7] | Screen-detected and interval cancer data, incidence observed from registry data | Maximum likelihood estimation | 45-74 | 4.9 (3.0 – 8.2) | 47 (30 – 65) |
| Launoy 1997  [7] | Screen-detected and interval cancer data, incidence observed from registry data | Prevalence to incidence ratio | 45-74 | 2.2 (1.9 – 2.7) | 75 (69 – 82) |
| Pinsky 2001  [16] | Screen-detected and interval cancer data, incidence observed from registry data | Maximum likelihood estimation (with adjustment for competing risks) | 45-74 | 4.6 (3.2 – 8.0) | 50 (29 – 70) |
| Pinsky 2001  [16] | Screen-detected and interval cancer data, incidence observed from registry data | Maximum likelihood estimation (with adjustment for competing risks and overdiagnosis) | 45-74 | 3.5 (2.6 – 4.9) | Not reported |
