# Supplementary material for: Quantifying the duration of the preclinical detectable phase in cancer screening: a systematic review
Source: Epidemiol Health. 2022 Jan 3;44:e2022008. doi: 10.4178/epih.e2022008 (PMC9117108; doi:10.4178/epih.e2022008)
Supplement: Supplementary Material 7. — Estimates of the preclinical detectable phase duration for breast cancer when screening included mammography only (Two-County trial, Nijmegen Screening Program, Florence District Screening Program and Ontario Breast Screening Program) with a description of the mathematical approach to estimation and model assumptions. [file epih-44-e2022008-suppl7.doc]

**Supplementary Material 7.** Estimates of the preclinical detectable phase duration for breast cancer when screening included mammography only (Two-County trial, Nijmegen Screening Program, Florence District Screening Program and Ontario Breast Screening Program) with a description of the mathematical approach to estimation and model assumptions.

| **Author, year** | **Data used** | **Type of mathematical model** | **Age range**  **(years)** | **Overall preclinical detectable phase duration in years (standard error or 95% confidence interval)** | **Test sensitivity in percentage (standard error or 95% confidence interval)** |
| --- | --- | --- | --- | --- | --- |
| **Two-County trial, Sweden** | | | | | |
| Paci, 1991  [25] | Interval cancer data, incidence estimated within the model | Regression of observed on expected | 40-49  50-59  60-69 | 1.2 (0.8 – 2.4)  4.3 (3.2 ― 6.6)  4.0 (3.1 – 5.7) | 69  91  88 |
| Duffy, 1995 | Interval cancer data, incidence estimated within the model | Regression of observed on expected | 70-74 | 2.8 (1.8 ― 5.9) | 71 |
| Chen, 1996  [27] | Screen-detected and interval cancer data, incidence observed from control group | Regression of observed on expected | 40-49  50-59  60-69 | 2.5 (2.1 – 2.9)  3.8 (3.4 ― 3.1)  4.2 (4.0 – 4.5) | 83 (74 ― 89)  100  100 |
| Chen, 1997  [28] | Screen-detected and interval cancer data, incidence observed from control group | Regression of observed on expected | 40-49  50-59  60-69 | 2.4 (2.1 – 2.9)  3.7 (3.4 ― 4.2)  4.2 (4.0 – 4.6) | 83 (76 – 91)  100  100 |
| Duffy, 1997  [29] | Screen-detected and interval cancer data, incidence estimated within the model | Regression of observed on expected | 40-49  50-59  60-69 | 2.4 (2.1 – 2.9)  3.7 (3.4 – 4.2)  4.2 (4.0 – 4.6) | 83 (76 – 91)  100  100 |
| Chen, 2000  [30] | Screen-detected cancer data, incidence estimated within the model | Regression of observed on expected | 50-59  60-69 | 3.3 (2.1 – 5.3)  3.8 (2.7 – 5.5) | Assumed 100  Assumed 100 |
| Shen, 2019  [23] | Screen-detected and interval cancer data, incidence estimated within the model | Maximum likelihood estimation | 40-49 | 3.0 2.9 1.0 | 70 80 90 |
| **Nijmegen Screening Program, the Netherlands** | | | | | |
| Straatman, 1997  [14] | Screen-detected cancer data, incidence estimated within the model | Maximum likelihood estimation | 35-49  35-65 | 1.35  2.22 | 92  100 |
| **Florence District Screening Program, Italy** | | | | | |
| Paci, 1991  [25] | Interval cancer data, incidence estimated within the model | Regression of observed on expected | 40-44  50-54  55-59  60-64  65-69  50-69 | 1.34 (0.74 – 7.02)  1.63 (0.97 – 5.04)  5.17 (3.44 ― 16.76)  4.68 (2.76 ― 14.36)  6.45 (3.88 – 18.99)  3.92 (3.01 – 5.82) | 72  88  86  91  95  89 |
| **Ontario Breast Screening Program, Canada** | | | | | |
| Jiang, 2016  [22] | Screen-detected and interval cancer data, incidence not included | Maximum likelihood estimation | 50-54 55-64 65+ | 3.6 (2.1 ― 5.0) 4.5 (3.2 ― 6.5) 4.4 (3.4 ― 9.1) | 93 (72 ― 100) 93 (78 ― 00) 99 (64 ― 100) |
