# Supplementary material for: Quantifying the duration of the preclinical detectable phase in cancer screening: a systematic review
Source: Epidemiol Health. 2022 Jan 3;44:e2022008. doi: 10.4178/epih.e2022008 (PMC9117108; doi:10.4178/epih.e2022008)
Supplement: Supplementary Material 8. — Estimates of the preclinical detectable phase duration for colorectal cancer in colonoscopy screening (German screening program, Germany) with a description of the mathematical approach to estimation and model assumptions. [file epih-44-e2022008-suppl8.doc]

**Supplementary Material 8.** Estimates of the preclinical detectable phase duration for colorectal cancer in colonoscopy screening (German screening program, Germany) with a description of the mathematical approach to estimation and model assumptions.

| **Author, year** | **Data used** | **Type of mathematical model** | **Age range**  **(years)** | **Overall preclinical detectable phase duration in years (95% confidence interval)** | **Test sensitivity in percentage (95% confidence interval)** |
| --- | --- | --- | --- | --- | --- |
| Brenner, 2011  [8] | Screen-detected cancer data, incidence observed from registry data | Prevalence to incidence ratio | Men, 55-59 Men, 60-64 Men, 65-69 Men, 70-74 Men, 75-79 Men, 80+ Women, 55-59 Women, 60-64 Women, 65-69 Women, 70-74 Women, 75-79 Women, 80+ | 5.5 (5.1 – 6.0) 5.2 (4.9 – 5.5) 4.7 (4.5 – 4.9) 4.9 (4.6 – 5.1) 5.0 (4.7 – 5.3) 5.5 (5.0 – 6.0) 4.7 (4.3 – 5.1) 4.5 (4.1 – 4.8) 4.6 (4.3 – 4.8) 4.8 (4.5 – 5.1) 5.2 (4.8 – 5.6) 5.8 (5.3 – 6.3) | Assumed 100 |

**References**

1.Hutchison GB, Shapiro S. Lead time gained by diagnostic screening for breast cancer. Journal of the National Cancer Institute 1968;41:665-681.

2.Zelen M, Feinleib M. On the theory of screening for chronic diseases. Biometrika 1969;56:601-614.

3.Shapiro S, Goldberg JD, Hutchison GB. Lead time in breast cancer detection and implications for periodicity of screening. American Journal of Epidemiology 1974;100:357-366.

4.Albert A, Gertman PM, Louis TA. Screening for the early detection of cancer. I. The temporal natural history of a progressive disease state. Mathematical Biosciences 1978;40:1-59.

5.Albert A. Screening for the early detection of cancer II. The impact of screening on the natural history of the disease. Mathematical Biosciences 1978;40

6.Louis TA, Albert A, Heghinian S. Screening for the early detection of cancer. III. Estimation of disease natural history. Mathematical Biosciences 1978;40:111-144.

7.Launoy G, Smith TC, Duffy SW, Bouvier V. Colorectal cancer mass-screening: Estimation of faecal occult blood test sensitivity, taking into account cancer mean sojourn time. International Journal of Cancer 1997;73:220-224.

8.Brenner H, Altenhofen L, Katalinic A, Lansdorp-Vogelaar I, Hoffmeister M. Sojourn time of preclinical colorectal cancer by sex and age: Estimates from the german national screening colonoscopy database. American Journal of Epidemiology 2011;174:1140-1146.

9.Walter SD, Day NE. Estimation of the duration of a pre-clinical disease state using screening data. American Journal of Epidemiology 1983;118:865-886.

10.Day NE, Walter SD. Simplified models of screening for chronic disease: estimation procedures from mass screening programmes. Biometrics 1984;40:1-14.

11.Brookmeyer R, Day NE, Moss S. Case-control studies for estimation of the natural history of preclinical disease from screening data. Statistics in Medicine 1986;5:127-138.

12.Brookmeyer R, Day NE. Two-stage models for the analysis of cancer screening data. Biometrics 1987;43:657-669.

13.Alexander FE. Estimation of sojourn time distributions and false negative rates in screening programmes which use two modalities. Statistics in Medicine 1989;8:743-755.

14.Straatman H, Peer PGM, Verbeek ALM. Estimating lead time and sensitivity in a screening program without estimating the incidence in the screened group. Biometrics 1997;53:217-229.

15.Shen Y, Zelen M. Parametric estimation procedures for screening programmes: Stable and nonstable disease models for multimodality case finding. Biometrika 1999;86:503-515.

16.Pinsky PF. Estimation and prediction for cancer screening models using deconvolution and smoothing. Biometrics 2001;57:389-395.

17.Hsieh HJ, Chen TH, Chang SH. Assessing chronic disease progression using non-homogeneous exponential regression Markov models: an illustration using a selective breast cancer screening in Taiwan. Stat Med 2002;21:3369-3382.

18.Pinsky PF. An Early- and Late-Stage Convolution Model for Disease Natural History. Biometrics 2004;60:191-198.

19.Shen Y, Zelen M. Robust modeling in screening studies: Estimation of sensitivity and preclinical sojourn time distribution. Biostatistics 2005;6:604-614.

20.Wu D, Rosner GL, Broemeling L. MLE and Bayesian inference of age-dependent sensitivity and transition probability in periodic screening. Biometrics 2005;61:1056-1063.

21.Cong XJ, Shen Y, Miller AB. Estimation of age-specific sensitivity and sojourn time in breast cancer screening studies. Statistics in Medicine 2005;24:3123-3138.

22.Jiang H, Walter SD, Brown PE, Chiarelli AM. Estimation of screening sensitivity and sojourn time from an organized screening program. Cancer Epidemiol 2016;44:178-185.

23.Shen Y, Dong W, Gulati R, Ryser MD, Etzioni R. Estimating the frequency of indolent breast cancer in screening trials. Stat Methods Med Res 2019;28:1261-1271.

24.Etzioni R, Shen Y. Estimating asymptomatic duration in cancer: the AIDS connection. Stat Med 1997;16:627-644.

25.Paci E, Duffy SW. Modelling the analysis of breast cancer screening programmes: Sensitivity, lead time and predictive value in the Florence District Programme (1975-1986). International Journal of Epidemiology 1991;20:852-858.

26.Duffy SW, Chen HH, Tabar L, Day NE. Estimation of mean sojourn time in breast cancer screening using a Markov chain model of both entry to and exit from the preclinical detectable phase. Statistics in Medicine 1995;14:1531-1543.

27.Chen HH, Duffy SW, Tabar L. A Markov chain method to estimate the tumour progression rate from preclinical to clinical phase, sensitivity and positive predictive value for mammography in breast cancer screening. Statistician 1996;45:307–317.

28.Chen HH, Duffy SW, Tabar L, Day NE. Markov chain models for progression of breast cancer. Part I: tumour attributes and the preclinical screen-detectable phase. J Epidemiol Biostat 1997;2:9-23.

29.Duffy SW, Day NE, Tabar L, Chen HH, Smith TC. Markov models of breast tumor progression: some age-specific results. Journal of the National Cancer Institute 1997;Monographs.:93-97.

30.Chen TH, Kuo HS, Yen MF, Lai MS, Tabar L, Duffy SW. Estimation of sojourn time in chronic disease screening without data on interval cases. Biometrics 2000;56:167-172.

31.Myles JP, Nixon RM, Duffy SW, Tabar L, Boggis C, Evans G, et al. Bayesian evaluation of breast cancer screening using data from two studies. Stat Med 2003;22:1661-1674.

32.Kim S, Jang H, Wu D, Abrams J. A Bayesian nonlinear mixed-effects disease progression model. J Biom Biostat 2015;6:271.

33.Shen S, Han SX, Petousis P, Weiss RE, Meng F, Bui AA, et al. A Bayesian model for estimating multi-state disease progression. Comput Biol Med 2017;81:111-120.

34.Freeman J, Hutchison GB. Prevalence, incidence and duration. Am J Epidemiol 1980;112:707-723.

35.Aarts AMWM, Duffy SW, Geurts SME, Vulkan DP, Otten JDM, Hsu CY, et al. Test sensitivity of mammography and mean sojourn time over 40 years of breast cancer screening in Nijmegen (The Netherlands). J Med Screen 2019;26:147-153.

36.Verbeek AL, Hendriks JH, Holland R, Mravunac M, Sturmans F. Mammographic screening and breast cancer mortality: age-specific effects in Nijmegen Project, 1975-82. Lancet 1985;1:865-866.
